# Radiation necrosis after a combination of external beam radiotherapy and iodine-125 brachytherapy in gliomas

**DOI:** 10.1186/s13014-021-01762-0

**Published:** 2021-02-23

**Authors:** Indrawati Hadi, Daniel Reitz, Raphael Bodensohn, Olarn Roengvoraphoj, Stefanie Lietke, Maximilian Niyazi, Jörg-Christian Tonn, Claus Belka, Niklas Thon, Silke Birgit Nachbichler

**Affiliations:** 1 Department of Radiation Oncology, University Hospital, LMU Munich, Marchioninistr. 15, 81377 Munich, Germany; 2 German Cancer Consortium (DKTK), Munich, Germany; 3 Department of Neurosurgery, University Hospital, LMU Munich, Munich, Germany

**Keywords:** Radiation necrosis, Stereotactic brachytherapy, External beam radiotherapy, Re-irradiation, Prognostic factors

## Abstract

**Purpose:**

Frequency and risk profile of radiation necrosis (RN) in patients with glioma undergoing either upfront stereotactic brachytherapy (SBT) and additional salvage external beam radiotherapy (EBRT) after tumor recurrence or vice versa remains unknown.

**Methods:**

Patients with glioma treated with low-activity temporary iodine-125 SBT at the University of Munich between 1999 and 2016 who had either additional upfront or salvage EBRT were included. Biologically effective doses (BED) were calculated. RN was diagnosed using stereotactic biopsy and/or metabolic imaging. The rate of RN was estimated with the Kaplan Meier method. Risk factors were obtained from logistic regression models.

**Results:**

Eighty-six patients (49 male, 37 female, median age 47 years) were included. 38 patients suffered from low-grade and 48 from high-grade glioma. Median follow-up was 15 months after second treatment. Fifty-eight patients received upfront EBRT (median total dose: 60 Gy), and 28 upfront SBT (median reference dose: 54 Gy, median dose rate: 10.0 cGy/h). Median time interval between treatments was 19 months. RN was diagnosed in 8/75 patients. The 1- and 2-year risk of RN was 5.1% and 11.7%, respectively. Tumor volume and irradiation time of SBT, number of implanted seeds, and salvage EBRT were risk factors for RN. Neither of the BED values nor the time interval between both treatments gained prognostic influence.

**Conclusion:**

The combination of upfront EBRT and salvage SBT or vice versa is feasible for glioma patients. The risk of RN is mainly determined by the treatment volume but not by the interval between therapies.

## Background

Despite of numerous improvements in the management of glioma, the treatments of patients with recurrent disease remain challenging. Various therapeutic options, such as surgery, re-irradiation and systemic therapy have been investigated in the last past years. The clinical decision depends usually on the pattern of relapse, prior treatments, age, and performance status of the patients. However, standard of care in patients with recurrence of gliomas is not yet well defined [1, 2].

The majority of gliomas relapse in field or adjacent to previously treated areas [3, 4], making the achievement of locoregional control more critical. Localized treatment strategies like re-irradiation have been considered increasingly in the past years [1]. External beam re-irradiation has been investigated in multiple retrospective studies and has been proven feasible and to improve outcome in selected patients [5, 6].

Besides external beam radiotherapy (EBRT), stereotactic brachytherapy (SBT) with iodine-125 seeds might offer a therapeutic alternative for glioma relapses in selected patients who previously underwent EBRT [7]. At our interdisciplinary neurooncology center, SBT using temporary iodine-125 seeds is routinely considered as an alternative treatment option for circumscribed, virtually spherical gliomas with a maximum diameter of 4 cm, which are not amenable for safe resection (due to eloquent or deep seated location), as an adjunct after incomplete resection, or in salvage situations [7–9]. The minimal-invasive, precise stereotactic implantation technique combined with a steep fall-off of the irradiation dose from the center of the tumor to the adjacent brain tissue makes SBT with iodine-125 an attractive therapeutic option for selected patients [9]. Its efficacy and feasibility in the primary setting have been described in some retrospective studies [8, 10–12]. For the recurrent setting, data of SBT after previous EBRT and vice versa are scarce. Therefore, we conducted a retrospective analysis of patients with glioma, who underwent upfront EBRT and salvage SBT or vice versa, to study the frequency of and the risk profile for radiation necrosis (RN) in glioma patients with this therapy combination.

## Patients and methods

### Patients

The tumor registry of the Departments of Neurosurgery and Radiation Oncology of the University Hospital of LMU Munich was queried for patients with glioma treated with low-activity, temporary iodine-125 SBT between 1999 and 2016. Only patients who had either additional upfront EBRT at first diagnosis or salvage EBRT due to progression after SBT were included in the analysis. Patient demographics were determined using patient`s electronic medical records and paper charts. Neuropathological diagnosis was done according to the 2007 WHO classification of central nervous system tumors [13]. Treatment parameters for EBRT as well as for SBT were collected and analyzed.

### Stereotactic brachytherapy planning

The technique of SBT has been described in detail previously [9]. In brief, after fusion of preoperative computerized tomography (CT) and magnetic resonance imaging (MRI) sequences and after 2007 co-localized dynamic positron emission tomography (PET) data, three-dimensional treatment planning was performed interdisciplinarily by the stereotactic neurosurgeon and the attending radiation oncologist. After outlining the tumor on the MRI slices the treatment plan was automatically calculated by the software (Brainlab, Target software, version 1.19; Brainlab AG, Munich, Germany). Temporary low-activity (≤ 21 mCi) iodine-125 seeds (model: OncoSeedtm IMC6711; Oncura Ltd., Austin, TX, USA) were encapsulated within the tip of a Teflon catheter, sterilized, stereotactically placed through a 2 mm burr hole, and secured. The correct position of the implanted seed(s) was checked with a CT scan on the following day. Dexamethasone was administered on the day of the procedure and tapered over the next 3 days. Overall hospitalization time for SBT was 4 days. Seed removal was carried out under local anesthesia.

### External beam radiotherapy planning

Patients were immobilized with a thermoplastic mask. For treatment planning, the acquired CT scans were usually fused with MRI scans and gross tumor volume (GTV), clinical target volume (CTV), and planning target volume (PTV) were delineated. Three-dimensional conformal treatment planning was performed with the Helax® TMS 6.1B1 (Nucletron, Veenendaal, The Netherlands) or the Oncentra® treatment planning system (OTP MasterPlan®, Nucletron, Solingen, Germany) and linear accelerators with a minimal nominal energy of 6 MV were employed. Radiotherapy was applied 5 days a week mostly with single fractions of 1.8–2.0 Gy once daily to a cumulative dose of 54.0–60.0 Gy. Other individual radiotherapy schemes were applied due to special circumstances.

### Biological effective doses (BED)

To compare both radiation modalities, the applied biologically effective doses (BED) were calculated using the linear quadratic model described by Dale [14]. This model allows the calculation of BED for protracted (SBT) and fractionated therapy (EBRT) and considers the repopulation factor as well. For each radiation modality, BED for late reacting tissue (BED Gy_10_) and tumor tissue (BED Gy_3_) were calculated separately. BED values of SBT were calculated at the boundary of the treatment volume. The formulas of the BED model are listed in Table 1. The drawback of the linear quadratic model by Dale is the calculation of BED values instead of equivalent dose in 2 Gy fractions (EQD2), which aggravated the comparison with others studies on re-irradiation. However, this model was the only method we could find to compare protracted and fractionated therapies.

**Table 1:** Formula by Dale describing the linear quadratic model

BED for fractionated therapy for late-reacting tissue	N × d × (1 + [d/3 Gy]) − K × T
BED for protracted therapy for late-reacting tissue	R × T × (1 + [2R]/[µ × 3 Gy]) − K × T
BED for fractionated therapy for tumor tissue	N × d × (1 + [d/10 Gy]) − K × T
BED for protracted therapy for tumor tissue	R × T × (1 + [2R]/[µ × 10 Gy]) − K × T

*BED* biologically effective dose (total dose × relative effectiveness − repopulation factor), *N* number of fractions, *d* fraction dose (Gy), *K* repopulation factor 0.6 (Gy × day^–1^), *T* treatment time (days), *R* dose rate (Gy × h^–1^), *µ* recovery half-life 0.46 (h ^–1^)

### Clinical follow-up and radiological assessment

Clinical assessment and MRI were performed in regular clinical follow-up visits every 3 (high-grade glioma, HGG) to 6 (low-grade glioma, LGG) months after irradiation. Any suspicious findings in the MRI were discussed in the interdisciplinary tumor board. Before 2008, stereotactic biopsy was the only certain method to differentiate between radiation necrosis and progressive disease. Metabolic imaging (18F-Fluoro-Ethyl-Tyrosine positron-emission tomography, FET-PET) was used in the clinical routine afterwards [15]. If the metabolic imaging failed to differentiate between radiation necrosis and progression, histological re-evaluation by stereotactic biopsy was performed. However, a histological proof of recurrence was always considered to be necessary by the interdisciplinary tumor board, before a salvage treatment could be done.

### Statistical analysis

Statistical analyses were done with IBM SPSS Statistics, Version 25 (IBM, Armonk, NY, USA). Patient demographics were calculated as absolute and relative frequencies. The Kaplan–Meier analysis was performed to assess radiation necrosis-free survival (RNFS). RNFS was defined as the interval between the date of second treatment modality and the date of radiation necrosis or the date of the last follow-up. The log-rank test and logistic regression models were used to assess the influence of various factors on radiation necrosis. A two tailed *p*-value of < 0.05 was considered significant. Furthermore, we determined optimal cut-off values for the identified risk factors using ROC and AUC analyses. To evaluate the correlation between radiation necrosis and local progression or overall survival, we performed Pearson’s chi-squared test for categorical variables. The institutional review board approved this analysis and all patients signed informed consent prior to the start of therapy.

## Results

### Study population

The study population comprised 86 patients (37 female, 49 male) with a median age of 47 years. At the time of first diagnosis, 38 patients suffered from histologically verified low-grade glioma (LGG) and 48 from high-grade glioma (HGG). Patients’ characteristics are summarized in Table 2. Malignant transformation was found in 28 patients afterwards, resulting in 13 patients with LGG and 73 patients with HGG at the time of second irradiation. Median follow-up after first diagnosis was 60 months and median follow-up after last irradiation was 15 months.

**Table 2:** Patient characteristics

Characteristics	Patients	
	Absolute (n)	Relative (%)
**Number of patients**	86	100
**Gender**		
Male	49	57
Female	37	43
**Age (years) median**	47 (range 18–77)	
**Histology at first diagnosis**		
Low-grade glioma	38	44
High-grade glioma	48	56
**Histology at second irradiation**		
Low-grade glioma	13	15
High-grade glioma	73	85
**Number of malignant transformations**	28	33
**Follow up (months) median**		
After first diagnosis	60 (range 7–353)	
After second irradiation	15 (range 0–167)	

There were 58 patients, who underwent an upfront EBRT at first diagnosis and salvage SBT; 28 patients were treated with upfront SBT and salvage EBRT. Treatment parameters are summarized in Table 3.

**Table 3:** Treatment parameters

Parameters	Total	
	Absolute (n)	Relative (%)
**Treatment sequence**		
Upfront EBRT	58	67
Upfront SBT	28	33
**SBT**	**Median (range)**	
Reference dose (Gy)	54.0 (20.0–60.0)	
Dose rate (cGy/h)	10.0 (5.0–22.0)	
Tumor volume (ccm)	3.56 (0.22–50.50)	
Treatment time (h)	500 (200–1044)	
BED for late reacting tissue (Gy_3_)	46.88 (17.40–72.89)	
BED for tumor tissue (Gy_10_)	41.59 (15.50–63.14)	
**Number of implanted seeds/patient**	**Number of patients**	
1	39	45
2	34	40
3	11	13
4	2	2
**EBRT**	**Median (range)**	
Treatment time (days)	43 (20–67)	
Dose of single fraction (Gy)	2.0 (1.60–2.67)	
Total dose (Gy)	60.0 (40.05–69.40)	
BED for late reacting tissue (Gy_3_)	73.6 (55.44–91.11)	
BED for tumor tissue (Gy_10_)	45.6 (34.27–54.49)	
**Cumulative BED after EBRT + SBT**	**Median (range)**	
Total BED for late reacting tissue (Gy_3_)	120.38 (83.73–180.92)	
Total BED for tumor tissue (Gy_10_)	87.23 (54.02–130.16)	
**Interval between EBRT and SBT (months)**	**Median (range)** 19 (2–227)	
**Systemic treatment simultaneous to re-irradiation**		
Yes	11	13
*Temozolomide*	*11*	*100*
No	69	80
Unknown	6	7
**Systemic treatment sequential after re-irradiation (within 1 year)**		
Yes	30	35
*Temozolomide*	*17*	*57*
*Temozolomide → Bevacizumab*	*1*	*3*
*Temozolomide → PCV*	*1*	*3*
*PC*	*1*	*3*
*PC → Bevacizumab/Irinotecan*	*1*	*3*
*Bevacizumab*	*2*	*7*
*Bevacizumab/Irinotecan*	*3*	*10*
*Bevacizumab/PC*	*1*	*3*
*Bevacizumab/Temozolomide*	*3*	*10*
No	49	57
Unknown	7	8
**Sequential bevacizumab after re-irradiation (within 1 year)**		
Yes	11	13
No	68	79
Unknown	7	8

EBRT: external beam radiotherapy; SBT: stereotactic brachytherapy; BED: biologically effective doses; PC: procarbazine, CCNU; PCV: procarbazine, CCNU, and vincristine

Median tumor volume of SBT was 3.56 ccm (0.22–50.50 ccm). One iodine-125 seed was used in 39 patients, two seeds in 34 patients, three seeds in 11 patients, and two patients were treated with 4 seeds. The tumors were irradiated with a median reference dose of 54.0 Gy (20.0–60.0 Gy) and a median dose rate of 10.0 cGy/h (5.0–22.0 cGy/h). The iodine-125 seeds were implanted temporarily for a median time of 500 h (200–1044 h). The median BED for late reacting tissue was 46.88 Gy_3_ (17.40–72.89 Gy_3_) and the median BED for tumor tissue was 41.59 Gy_10_ (15.50–63.14 Gy_10_).

EBRT was performed with a median single fraction dose of 2.0 Gy (1.60–2.67 Gy) and a median total dose of 60 Gy (40.05–69.40 Gy). The median duration of EBRT was 43 days (20–67 days). The late reacting tissue received a median BED of 73.6 Gy_3_ (55.44–91.11 Gy_3_) and the tumor received a median BED of 45.6 Gy_10_ (34.27–54.49 Gy_10_).

After the combination treatment with SBT and EBRT, the median overall cumulative BED for late reacting tissue was 120.38 Gy_3_ (83.73–180.92 Gy_3_) and the median cumulative BED for tumor tissue was 87.23 Gy_10_ (54.02–130.16 Gy_10_). The median interval between EBRT und SBT was 19 months (2–227 months). During the second radiation treatment, 11 patients (12.8%) received simultaneous temozolomide. Within 1 year after the re-irradiation, 30 patients (34.9%) received systemic therapy. Among these 30 patients, 11 patients were treated sequentially with bevacizumab. Treatment parameters are summarized in Table 3.

### Outcome

At the time of last follow-up, 37 patients had developed local progression and 69 patients were dead.

**Fig. 1:**
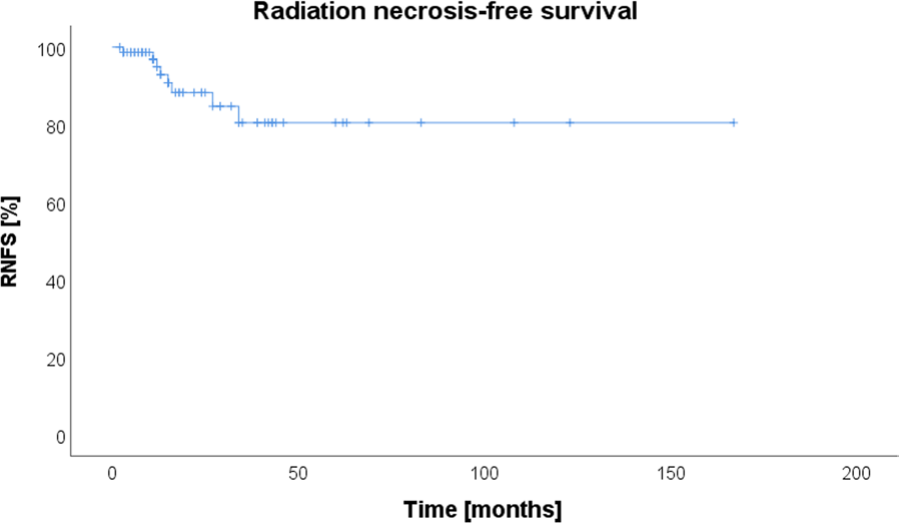
Kaplan–Meier estimates of radiation necrosis-free survival (RNFS). At the time of last follow up, radiation necrosis was diagnosed in 8 out of 75 patients (10.7%)

Radiation necrosis was diagnosed in 8 of 75 patients (10.7%) (Fig. 1). Seven out of these 8 patients with RN described deterioration of neurological deficits and therefore they had to be treated with dexamethasone. One patient developed steroid refractory RN and had to be treated with bevacizumab. Three of the 8 patients (37.5%) with RN developed local progression, there was no significant correlation between RN and local progression (*p* = 0.504). Seven of the 8 patients (87.5%) with RN were dead at the time of last follow-up. There was no significant correlation between RN and overall survival (*p* = 0.575). Due to lack of clinical and radiological assessment data, existence of radiation necrosis could not be determined in 11 patients (9 patients are deceased according to registration office data, 2 patients are from abroad). Estimated 1-year radiation necrosis-free survival (RNFS) was 94.9% and 2-year RNFS was 88.3%.

**Fig. 2:**
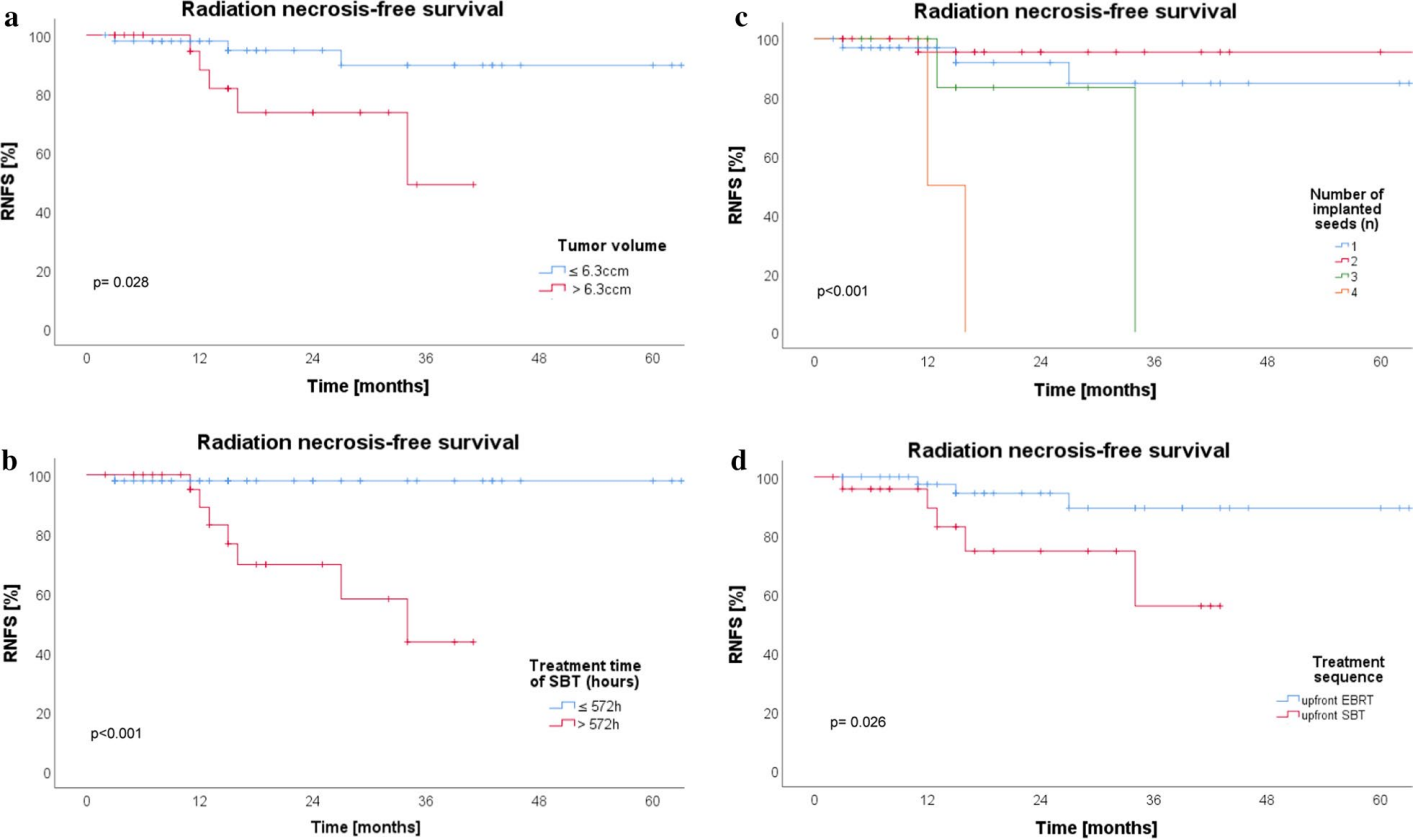
Tumor volume at stereotactic brachytherapy (SBT) was significantly associated with radiation necrosis-free survival (RNFS) (a) (> 6.3ccm**,** *p* = 0.028). Furthermore, irradiation time of SBT (b) (> 572 h, *p* < 0.001), the number of implanted seeds (c) (*p* < 0.001), and initial SBT before salvage EBRT (d) (*p* = 0.026) were significant risk factors for occurrence of radiation necrosis

In univariate analyses, tumor volume at SBT (> 6.3 ccm) was significantly associated with occurrence of radiation necrosis (HR 4.43, 95%-CI: 1.04–18.90, *p* = 0.028) (Fig. 2a). Furthermore, irradiation time of SBT > 572 h (HR 17.05, 95%-CI: 2.08–139.79, *p* < 0.001), number of implanted seeds (HR 2.67, 95%-CI: 1.22–5.76, *p* < 0.001), and initial SBT before salvage EBRT (HR 4.44, 95%-CI: 1.05–18.79, *p* = 0.026) were significant risk factors for radiation necrosis (Fig. 2b–d). Regarding therapy sequences, among the 58 patients who received upfront EBRT, 3 patients (5.2%) developed RN. Meanwhile 5 of the 28 patients (17.9%) who were treated with upfront SBT developed RN. Neither of the BED values gained prognostic influence. Other parameters such as gender, histology (initial and at the time of second irradiation) and malignant transformation were not significant. Also sequential bevacizumab after re-irradiation was not significantly associated with occurrence of RN. The time interval between EBRT and SBT (*p* = 0.363) was not associated with an increased risk of radiation necrosis. The results of the univariate analyses for prognostic factors for radiation necrosis are shown in Table 4.

**Table 4:** Univariate analysis

Radiation necrosis		
Parameters	Univariate analysis	
	HR (95% CI)	*p*-value
Gender (♂ vs. ♀)	0.82 (0.20–3.29)	0.780
Histology at first diagnosis (*LGG vs. HGG*)	1.48 (0.35–6.22)	0.590
Histology at second irradiation (*LGG vs. HGG*)	0.28 (0.06–1.41)	0.124
Malignant transformation (*yes vs. no*)	5.51 (0.68–44.98)	0.111
Treatment sequence (*upfront SBT vs. upfront EBRT*)	4.44 (1.05–18.79)	0.026*
**SBT**		
Reference dose > 52 Gy	2.11 (0.50–8.84)	0.307
Dose rate > 12 cGy/h	0.19 (0.02–1.53)	0.119
Tumor volume > 6.3 ccm	4.43 (1.04–18.90)	0.028*
Treatment time > 572 h	17.05 (2.08–139.79)	< 0.001*
**Number of implanted seeds/patient**		
> 2 seeds	2.67 (1.22–5.76)	< 0.001*
**EBRT**		
Treatment time > 43.5 days	1.46 (0.36–5.84)	0.595
Dose of single fraction > 1.8 Gy	0.37 (0.09–1.47)	0.156
Total dose > 60.0 Gy	0.04 (0.00–3118.9)	0.581
**Cumulative BED after EBRT + SBT**		
Total BED for late reacting tissue > 120 Gy_3_	0.13 (0.02–1.03)	0.053
Total BED for tumor tissue > 87 Gy_10_	0.13 (0.02–1.03)	0.053
Interval between EBRT and SBT	0.99 (0.96–1.01)	0.363
Sequential bevacizumab after re-irradiation—within 1 year (*yes vs. no*)	0.04 (0.00–637.8)	0.307

## Discussion

The management of recurrent gliomas remains challenging. Beside surgery and chemotherapy, re-irradiation has been increasingly considered and performed in the last years [1, 16]. In terms of re-irradiation, there is some heterogeneity regarding the modality of re-irradiation and the applied doses. Several approaches such as stereotactic radiosurgery (SRS), (hypo)fractionated stereotactic radiotherapy (FSRT), and also brachytherapy have been conducted [17]. Not to mention, the different technical solutions like linear accelerator, tomotherapy etc., and different types of radiation (e.g. photons, protons etc.).

Since re-irradiation of gliomas often becomes unavoidable, it raises the question of side effects, particularly radiation necrosis. Furthermore, it is a challenging task to differentiate between radiation necrosis and tumor progression after previous (chemo)radiotherapy. Melguizo-Gavilanes et al. reported that the concordance between radiological interpretation of MRI scans and subsequent histological diagnosis was reached in only 32% of the cases. This result showed that MRI scan is not a reliable method to detect pseudoprogression [18, 19]. Metabolic imaging, like 18F-FET-PET is an attractive method in differentiation between pseudoprogression and tumor recurrence with higher specificity and sensitivity compared to MRI. Still, this method is considered as an addition instead of replacement of stereotactic biopsy or close follow-up [20].

Our study showed a radiation necrosis rate of 10.7% after the therapy combination of SBT and EBRT. Tumor volume is the most important prognostic factor in our study and is significantly associated with the occurrence of radiation necrosis. Other prognostic factors were irradiation time of SBT and the number of implanted seeds. These two factors are related to tumor volume, since the number of implanted iodine-125 seeds and the duration of irradiation time of SBT increase with larger tumor volume. The therapy sequence was a prognostic factor as well; patients with initial SBT had a significantly higher risk of radiation necrosis development than patients who underwent initial EBRT. Presumably, patients with upfront SBT had a smaller tumor volume at first treatment and a larger recurrence, so it was impossible to undergo a second SBT in the relapse situation. Patients with upfront EBRT and salvage SBT had supposedly a smaller recurrence, which was possible to be treated with SBT. For EBRT, it is well known that 5% and 10% risk of radiation necrosis occurred at a BED of 120 Gy_3_ (range 100–140 Gy_3_) and at a BED of 150 Gy_3_ (range, 140–170 Gy_3_) [21]. Kong et al. reported a RN rate of 24% and Imber et al. of 16% after EBRT with 60 Gy in 2 Gy fractions and a single fraction re-irradiation with 16 Gy. This is equal to a summed EQD2 of 120 Gy and a BED of 200 Gy_3_ for late reacting tissue [22, 23]. Based on historical data, radiogenic complications after SBT alone occurred in 9% for tumors with a diameter < 4 cm and higher complication rates (up to 25%) were reported for tumors harboring larger diameters (≥ 4 cm) [9]. Hence, our analysis showed that the combination of SBT and EBRT in small circumscribed tumors slightly increased the risk for RN compared to SBT or EBRT alone. Unfortunately, there was limiting data regarding threshold for radiation necrosis after sequential EBRT and SBT. In the current analysis, the median overall cumulative BED for late reacting tissue was 120.38 Gy_3_ (83.73–180.92 Gy_3_) and the median cumulative BED for tumor tissue was 87.23 Gy_10_ (54.02–130.16 Gy_10_). Neither of the BED values gained prognostic influence presumably due to the homogeneity of the performed therapies and consequently BED values in all patients. A threshold of BED values for radiation necrosis could not be defined in our study. The time interval between EBRT and SBT (*p* = 0.363) was not associated with an increased risk of radiation necrosis.

Majdoub et al. reported similar findings, which described SBT as therapeutic option for patients with glioma. They performed a retrospective analysis of 63 patients with oligodendroglioma WHO II and WHO III, who were treated with SBT either as primary, adjuvant after incomplete resection or as salvage therapy after recurrence. It showed that SBT achieved comparable control rates to surgery and radio-/chemotherapy with a low rate of side effects. Only 11 patients (17%) suffered from temporary treatment related morbidity (nausea/vomiting, mild left-sided hemiparesis, and headache). The symptoms were reversible within 6 weeks under steroids. Radiation necrosis was not described. However, only 14 out of 63 patients (22%) were treated with SBT after previous EBRT [12].

A comparable study to our data is an analysis from Romagna et al., who compared SBT as upfront (n = 20 patients) and salvage treatment (n = 28 patients) for small brain metastases. The patients in the salvage group underwent previous WBRT alone (median cumulative dose 35 Gy, 8 patients), WBRT in combination with a stereotactic tumor boost (median boost dose 18 Gy, 7 patients), or SRS alone (median dose 18 Gy, 8 patients). The median tumor volume was 3.4 ml, which is comparable to our data. The median overall cumulative BED for late-reacting tissue was 180.7 Gy_3_ in the salvage group, which was higher than our cumulative BED for late-reacting tissue (median 120.38 Gy_3_)_._ With a follow-up of 15 months after the last irradiation treatment, transient symptomatic edema was found in 2 patients receiving a cumulative BED > 190 Gy_3_. Because of the low number of adverse events, a threshold of BED values for radiogenic complications could not be identified. This study underscored that SBT is a feasible therapeutic option despite previous irradiation [8]. Ruge et al. also reported the safety of WBRT combined with low-dose SBT, there was no RN reported in their study [24].

Kickingereder et al. reported about 201 patients with glioblastoma (GBM), who were treated with a combination of SBT and EBRT [25]. Ninety-eight patients underwent EBRT with 60 Gy at first diagnosis and SBT at recurrence, whereas 103 patients with inoperable GBM were treated with SBT and EBRT boost as primary treatment. Symptomatic RN was found in 3 patients (1.5%) within 3–9 months after SBT with tumor volume being a risk factor for RN development. This study supports low-dose-rate SBT to be a safe treatment option for inoperable primary glioblastomas as well as recurrences. Similar findings were reported by Suchorska et al.. Among 172 patients with WHO grade III glioma, 66 patients were treated with low-dose-rate SBT after previous EBRT. Only 2/172 patients developed permanent radiogenic morbidity and transient edema was found in 22/172 patients, which improved under steroids in 21 patients. RN was not mentioned explicitly, but overall the complication rate was considerably low [26].

Besides low-dose-rate (LDR) SBT as we and the above mentioned studies used, high-dose-rate (HDR, 30–50 cGy/h) SBT combined with EBRT was applied especially in ancient studies. Three prospective trials showed that a HDR-SBT boost in addition to EBRT for malignant glioma failed to improve survival but resulted in high complication rates, including brain edema, radiation necrosis, and need for re-operation for clinical deterioration in up to 50% [27–29].

As mentioned above, another approach of re-irradiation after previous EBRT is to perform a second EBRT. Heterogeneous dose and fractionation schemes were reported in previous studies, such as normofractionated conventional radiotherapy (e.g. 36 Gy in 18 to 20 fractions) or FSRT (e.g. 30 Gy in 6 fractions or 25 Gy in 5 fractions) [5, 6, 30–32]. Salvage re-irradiation was well tolerated with radiation necrosis rates ranging from 0 to 11% [5, 6, 30–32].

Another option of performing re-irradiation is to apply a high dose in one fraction using SRS. A systematic review of 29 studies showed SRS to be a relatively safe treatment in the salvage situation with a radiation necrosis rate of 5.9% (0–44%). However, the relatively low rate of RN depends on the tumor volume. Hall et al. analyzed 35 patients with HGG and a large tumor volume (median 28 cm^3^), who were treated with SRS. In seven patients surgical resection had to be performed due to increasing mass effect 4.0 months (mean) after SRS, resulting in an actuarial re-operation rate of 31% [33].

Our study has some limitations. Firstly, because of its retrospective nature, we could not determine radiation necrosis in 11 patients due to lack of clinical follow-up and radiological assessment. Furthermore, only patients with clinically symptomatic radiation necrosis were considered in our analysis. The limitation of SBT itself that seeds can only be implanted in small circumscribed tumors leads to a selection bias.

Nevertheless, since the therapy management of recurrent glioma is still limited and challenging, the combination of EBRT and SBT with iodine-125 seeds revealed to be a safe therapeutic option.

## Data Availability

The datasets used and analysed during the current study are available from the corresponding author on reasonable request.
